# Evaluation of Screen Time in Children Under Five Years Old

**DOI:** 10.7759/cureus.54444

**Published:** 2024-02-19

**Authors:** Nese Mutlu, Meltem Dinleyici

**Affiliations:** 1 Department of Pediatrics, Eskişehir Osmangazi University Faculty of Medicine, Eskişehir, TUR; 2 Department of Social Pediatrics, Eskişehir Osmangazi University Faculty of Medicine, Eskişehir, TUR

**Keywords:** screen time, smartphone, computer, tablet, television, media, child

## Abstract

Introduction: Due to the rapid advancement of technology, there has been a noteworthy increase in the diversity and abundance of activities involving children. The most effective methods to enhance and facilitate children's media interactions are to minimize, reduce, use with caution, and establish healthy patterns. We aimed to evaluate media exposure of children below five years of age.

Material and methods: This is a prospective, observational, cross-sectional study that was conducted between December 2017 and September 2019 in Eskişehir, Türkiye. To assess the frequency of electronic device usage among children under the age of five, including televisions, laptops, tablets, and mobile phones, as well as its impact on their sleep patterns and physical measurements, and to evaluate families' understanding of the terms "screen time" and "back screen time," we developed a questionnaire.

Results: We analyzed a total of 731 questionnaires: 334 (45.7%) were girls, 397 (54.3%) were boys, and the mean age was 33.55±16.37 months. Upon examining the technical equipment accessible to the children in our study, we found that 98.6% possessed a television, 96.9% owned a mobile phone, 54% had a laptop, 49.5% had a tablet, and 34.1% possessed a gaming console. The study revealed the following proportions of electronic devices in children's rooms: 13% televisions, 11.9% tablets, 7.4% laptops, and 7% mobile phones. There has been a substantial increase in the amount of time they spend watching television and playing computer games among children who have at least one sibling. There was a statistically significant disparity between the television viewing periods and the body mass index of children older than two years old. Additionally, we have seen a significant disparity in the presence of media devices in children's bedrooms and the subsequent impact on their sleep duration and patterns throughout both nighttime and daytime. Around 65.8% of parents did not know of the concept of screen time, while 88.4% of parents did not know of the concept of back screen time.

Discussion: Parental compliance with the current guidelines for screen time is insufficient, among parents with children under the age of five, even though exposure to screens begins in the first months of life. Our analysis highlighted the necessity for parents to establish and enforce a unified and logical media usage policy for all children residing in the household. It is crucial to allocate sufficient time during the routine healthcare visit to discuss these recommendations.

## Introduction

Technological gadgets, such as smartphones, tablets, and computers, constantly surround individuals of all ages in various settings, including their homes, schools, and workplaces [1]. As technology continues to progress quickly, the number of activities involving children is also increasing, and the nature of these activities is evolving. These technologies have become fundamental components that influence children's interaction with their surroundings, their comprehension of amusement, and their education [2]. Children often encounter and acquire proficiency in using modern items such as televisions, smartphones, computers, and tablets in their surroundings [3].

The socioecological model, as proposed by Kaur and colleagues, offers an explanation for the factors associated with screen use in children under the age of five. The child's own characteristics (biological, behavioral, and demographic), the child's media environment (type of child care, rules regarding media access), and the child's broader sociocultural environment (location, laws, and time of year) influence the caregiver's characteristics (biological, behavioral, and demographic) in this inverted model [4]. The family's opinions, attitudes, and beliefs about media culture influence the screen time of children [1,5]. Research findings revealed a positive correlation between children's screen time and being the eldest child, having a working mother, and an overall rise in the family's screen time [1,6-10]. There is a negative correlation between children's screen time and family income, socioeconomic status, education, and physical activity [9-12].

Factors such as children's absence from school or childcare, prolonged exposure to television as a background activity, and eating meals while watching television all contribute to the issue [13,14]. Research suggests that toddlers below the age of two do not derive any scholastic advantages from media exposure [15]. Children in this age group can improve their language skills by watching educational videos with their family, since it encourages cooperative learning among them. Studies have shown that toddlers aged two can improve their early learning by interacting with touch-screen gadgets and e-book applications that provide responsive feedback, enabling them to learn letters, phonetics, and words [16-18].

Over a span of nearly 50 years, researchers have consistently shown that media studies affect the health of children and adolescents, regardless of the type of technology used or the appropriateness of the material [19,20]. Regardless of whether old or new technologies are utilized or whether they are used appropriately or with suitable material, this impact remains consistent [20]. Lin et al. [21] revealed a significant correlation between the duration of television viewing and the enhancement of cognitive, language, and motor abilities in infants between the ages of 15 and 35 months. Many factors were found to have significant correlations with their respective effects, including unhealthy eating habits; increased body mass index (BMI) and risk of obesity; late-onset eating disorders; language, motor skill, and cognitive development; decline in academic success; parent-child conflict; aggressive and/or antisocial behavior; mood or hyperactivity disorder; and sleep issues [1,2,8,13,21-29].

The most effective methods to enhance and facilitate children's media interactions are to minimize, reduce, use with caution, and establish healthy patterns [30]. The American Academy of Pediatrics (AAP) and the Australian Health Department recommend that children below the age of two should not have any exposure to screens. These rules entail refraining from using electronic devices during meals, abstaining from screen usage for at least one hour prior to bedtime, avoiding the use of screens to pacify or soothe children outside of the home, avoiding exposure to low-quality television shows or movies, and abstaining from programming that contains violence or content that may cause distraction. Children between the ages of two and five should limit their daily television viewing to a maximum of one hour, while children aged six to eight should limit their television viewing to a maximum of two hours. It is advisable for families to oversee their children's media intake and app downloads while their children, aged two to eight, are using screens in order to guarantee that their children are deriving educational benefits from these activities. It emphasizes the significance of families together choosing media that is suitable for the age of their children [31]. Nonetheless, implementing these proposals poses several obstacles. In Türkiye, our previous study showed knowledge regarding the use of old and new media is limited among the parents and screen time and mobile device use (including during meals) are common in children below two years of age [32]. In this study, we considered the potential correlation between the increasing frequency of screen addiction and prolonged exposure to screens starting from a young age. This research aimed to determine the knowledge of parents who have children under five years of age about media use at home. We aimed to evaluate the potential relationship between child media exposure and family socioecological status, children's anthropometric measurements and the presence of overweight or obesity, and also children's sleep schedules.

## Materials and methods

Between December 2017 and September 2019, we conducted an observational, cross-sectional study in the Pediatric Department of Eskişehir Osmangazi University Faculty of Medicine in Eskişehir, Türkiye. All procedures performed in studies involving human participants were in accordance with the ethical standards of the institutional and/or national research committee and with the 1964 Helsinki Declaration and its later amendments or comparable ethical standards. The Local Ethics Committee of Eskişehir Osmangazi University Faculty of Medicine approved the study on November 21, 2017 (approval number: 5).

We developed a questionnaire form based on the pertinent literature and used it as a tool for data collection. We applied the questionnaire forms to the families by asking them to fill them out face-to-face or individually and obtaining their consent. The mother or father signed the consent forms. The questionnaire comprises a total of 29 questions, divided into two sections. The initial section comprises the child's identifying data, the family's sociodemographic data, and the child's anthropometric measures. The health status of the mothers and fathers was noted according to their self-reports. Assessments were mostly focused on the presence of physical illnesses. In the second part, we examine the household environment where the family resides, including the presence and quantity of televisions, computers, mobile phones, and tablets in the home and specifically in the child's room. Additionally, we analyze the amount of time both children and their parents spend using these screens, the content they consume, the level of parental control over their child's screen exposure, and the children's bedtime routine. The questionnaire includes open- and closed-ended multiple-choice questions regarding sleep patterns during the day and night, the presence of visual impairments, attendance at kindergarten or nursery, understanding of screen orientation and usage, and prior knowledge about these topics. Parents were requested to indicate the amount of time their children spend viewing screens and engaging with tablets and computers using the following categories: never, 30 minutes to one hour, one to two hours, three to four hours, and more than five hours.

This study aimed to evaluate the frequency at which televisions, laptops, tablets, and mobile phones are present in households and children's rooms, as well as the usage patterns of these media devices among families and their children under the age of five. The purpose was also to assess the correlation between children and families and their sociodemographic features, as well as investigate the impact of children on their sleep patterns and length. Additionally, the study aimed to assess the families' understanding of the notions of "screen time" and "back screen time," as well as their knowledge of these ideas as conveyed by their physicians. Additionally, the study aimed to investigate the families' screen usage patterns for their children and the extent of their control during such periods. The study determined the screen front and rear screen front times of children under the age of five, the information status related to the screen front and rear screen front times of the families, the relationship between the sociodemographic findings of the families and the screen time of the children, and the anthropometric measurements of children and the screen with BMI. We planned to evaluate the relationship between time and the sleep time of children.

We performed the statistical analyses of the data obtained within the scope of the study using IBM SPSS Statistics for Windows, Version 21.0 (Released 2012; IBM Corp., Armonk, New York, United States). We report continuous data as mean±standard deviation (mean±SD). Categorical data are defined as n%. The suitability of the data for a normal distribution was tested with the Shapiro-Wilk test. We used the Pearson, chi-squared, and Fisher exact tests to analyze the categorical data. We used a one-way analysis of variance (ANOVA) test to compare the groups. We applied the multiple comparison test (according to Bonferroni correction) to determine the effect of the difference in cross tables with statistically significant differences in categorical data. The statistical significance level was determined to be p<0.05.

## Results

We surveyed a total of 855 parents who applied to the Pediatric Department of Eskişehir Osmangazi University Faculty of Medicine between December 2017 and September 2019 to gather the research data. Parental non-participation, partial responses, and overall incompleteness resulted in the exclusion of 124 questionnaires from the study. As a result, we analyzed a total of 731 questionnaires. Out of the total number of children who participated in the study, 334 (45.7%) were girls, and 397 (54.3%) were boys. The mean age of all groups was 33.55±16.37 (range: 2-59) months. Considering the age groups of children in the study group, 105 children (14.4%) are between one and 12 months, 141 children (19.3%) between 13 and 24 months, 133 children (18.2%) between 25 and 36 months, 178 children (24.4%) between 37 and 48 months, and 173 children (23.7%) between 49 and 60 months. Out of the children involved in the study, 270 (36.9%) are only children, while 461 (63.1%) have at least one sibling (Table 1).

**Table 1:** The demographic, educational, health, and socioeconomic findings of the study group

	n (%)
Gender (girls/boys)	334/397
Age groups (months)	
1-12 months	105 (14.4%)
13-24 months	141 (19.3%)
25-36 months	133 (18.2%)
37-48 months	178 (24.4%)
49-60 months	173 (23.7%)
Presence of sibling	461 (63.1%)
Child caregiver	
Mother	573 (78.4%)
Father	7 (0.9%)
Grandparents	114 (15.6%)
Others	37 (5.1%)
Mother's educational status	
Elementary and secondary school	181 (24.7%)
High school/college	247 (33.8%)
University and graduate	303 (41.5%)
Father's educational status	
Elementary and secondary school	171 (23.4%)
High school/college	267 (36.6%)
University and graduate	293 (40%)
Mothers: previously healthy	702 (96%)
Fathers: previously healthy	699 (95.6%)
Caregivers: previously healthy	669 (91.6%)
The family has sufficient money to meet their expenses	488 (66.8%)

The mean age of mothers is 31.9±5.1 (range: 19-48) years; fathers have a mean age of 35.2±5.3 (range: 23-59) years; and caregivers have a mean age of 36.3±10.7 (range: 19-73) years. Mothers are responsible for the care of 573 children (78.4%), and fathers, on the other hand, provide care for just seven children (0.9%). Grandparents take care of 114 children (15.6%), while other individuals are responsible for the care of 37 children (5.1%). The educational and health status of parents and caregivers have been summarized in Table 1.

Upon analysis, it was found that 66.8% of the 488 families have sufficient money to meet their expenses, while 22% of the 161 families lack enough income to cover their expenses (Table 1). Among the 194 (26.5%) children that attend nursery school, 89.6% of them are over the age of two. The proportion of university and graduate mothers sending their children to nursery and kindergarten was significantly greater compared to primary, secondary, and high school graduates (p<0.05). The study revealed a substantial decrease in the participation rates of children from households with insufficient resources to cover their expenses (p<0.05).

The average age at which children began consuming complementary foods was 5.91±1.57 months. Regarding the anthropometric measurements of children involved in the study, 43 children (5.9%) fall below the third percentile. When we assessed the children based on the ratio of body weight to height, we diagnosed 182 children (24.9%) with protein-energy malnutrition, classified 510 children (69.8%) as normal, and identified 39 children (5.3%) as obese.

**Figure 1:**
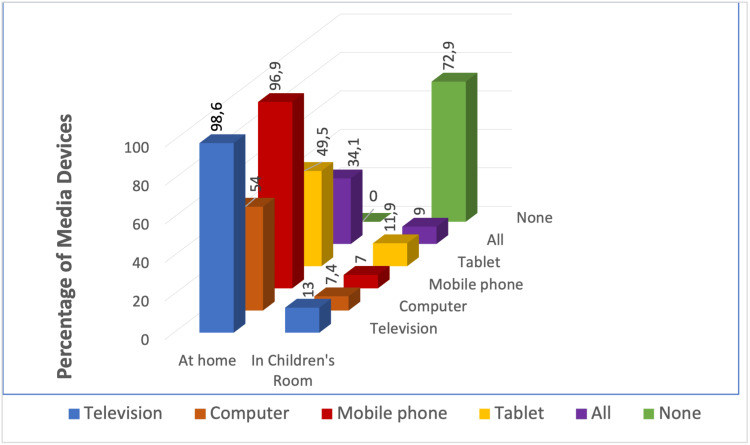
Percentage of media devices at home and in children's rooms

Out of the total population surveyed, 721 individuals (98.6%) reported having a television at home, 708 individuals (96.9%) reported owning a mobile phone, 395 individuals (54%) reported having a computer, 362 individuals (49.5%) reported owning a tablet, and 249 individuals (34.1%) reported having all three of these devices in their family. Among the children's rooms surveyed, the majority (72.9%) did not have any media tools. However, 13% of the rooms had televisions, 11.9% had tablets, 7.4% had computers, and 7% had mobile phones. All media tools were present in only 1.2% of the rooms (Figure 1).

When surveyed about the presence of televisions in different rooms, the results are as follows: 93.3% of the 682 residences have televisions in the living room, 26.8% have televisions in the kitchen, 7.7% have televisions in the children's room, 6% have televisions in the parent's room, and 8.8% have televisions in other areas such as the living room or balcony. When asked about the number of mobile phones in households, 509 (69.4%) had two, 131 (17.9%) had three, 53 (7.3%) had four, and 21 (2.9%) had five or more.

After investigating the ownership of mobile phones among all children residing in the households, we discovered that 98 children (13.4%; mean 12.5±3.4 years) had mobile phones, while 633 children (86.6%) did not. We identified four youngsters who were under the age of five.

**Figure 2:**
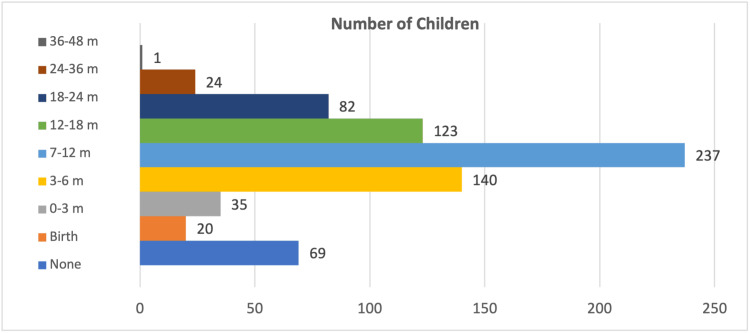
Distribution of children according to the age at which they started watching television

When questioned about the appropriate age for children to begin viewing television in the study, the largest group, consisting of 237 individuals (32.4%), indicated that one year or later was the recommended age. Subsequently, 140 families (19.2%) responded after six months, 123 families (16.8%) after 1.5 years, 82 families (11.2%) after two years, 69 families (9.4%) never, 35 families (4.8%) after three months, 24 families (3.3%) after three years, 20 families (2.7%) after birth, and one family (0.1%) after four years of age. Around 32.4% of all children initiated television viewing at the age of one. There was no statistically significant correlation observed between the timing of introducing additional meals and the age at which children began viewing television (p>0.05) (Figure 2).

**Figure 3:**
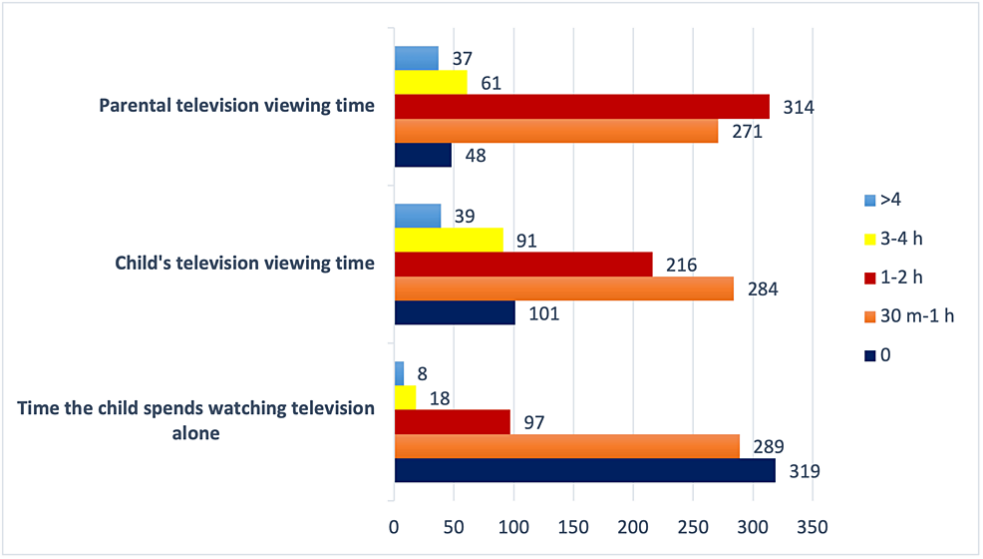
Children's and parents' television viewing time

Upon analysis, we discovered that 314 parents, which accounted for 43% of the total, spent one to two hours watching television daily. During other monitoring periods, we found that 271 families (37.1%) spent 30 minutes to one hour watching television, 61 families (8.3%) spent three to four hours, and 37 families (5.1%) spent more than five hours. Forty-eight households (6.6%) reported that they abstained from watching television. Upon examination, we discovered that the majority of children (284 children, 38.9%) watched television for a duration of 30 minutes to one hour. The remaining 216 children (29.6%) had viewing times of one to two hours, 91 children (12.4%) watched television for four hours, and 39 children (5.3%) watched television for a duration exceeding five hours. A total of 101 children (13.8%) abstain from watching television. Among the children surveyed, 69 (47.3%) of them, who are under the age of two, spend at least 30 minutes viewing television (Figure 3).

When analyzing the correlation between the television viewing habits of families and their children, we found that families who do not watch television have significantly higher rates of children who also do not watch television, compared to families who watch television for at least 30 minutes (p<0.05). We conducted an investigation to determine if the presence of at least one sibling impacted the amount of time children spent watching television. There has been a substantial increase in the amount of time they spend watching television among children who have at least one sibling (p<0.05). The mother's educational attainment influences the amount of time spent on screens. Specifically, a greater proportion of high school graduate mothers reported never watching television compared to mothers who completed primary or secondary school (p<0.05). The proportion of mothers who graduated from primary school and watched more than five hours of television was much greater than the proportion of mothers who graduated from middle school, high school, and university (p<0.05). However, there was no significant correlation observed between the father's educational position and the children's television viewing habits (p>0.05).

The majority of participants responded with "cartoons" when asked open-ended questions about the content children see on television. The majority of families, 493 (67.4%), selected news programs, series, and music channels as their preferred viewing options when asked multiple-choice questions about their preferences. A total of 631 families said that their children were present when they watched television.

There was no statistically significant correlation found between the amount of time children under two years of age spent watching television and their body fat percentage (p>0.05). Children over two years of age showed a statistically significant difference in their average BMI based on their television viewing times (p<0.05). Upon investigation of the potential correlation between visual impairments in children and the amount of time spent watching television, it was found that out of the 44 children (6%) with visual defects, there was no statistically significant association between their impairments and the duration of television viewing (p>0.05).

A study found that the length of time children spend engaging in computer and tablet games is consistent across all age groups and most children do not play these games at all. While 613 (83.9%) children never play with a computer, 55 children (7.5%) play with the computer for 30 minutes to one hour, 42 children (5.8%) play with the computer for one to two hours, 17 children (2.3%) play with the computer for three to four hours, and four children (0.5%) play with the computer for more than five hours (Table 2). Researchers investigated whether the presence of a sibling affects the duration of computer game playing among children. The results revealed a statistically significant increase in the number of individuals without siblings in the group that never played games, compared to those without siblings in the group that played for one to two hours (p<0.05). There was no statistically significant correlation observed between the education of the mothers and fathers and the amount of time the children spent playing computer games (p>0.05). Researchers posed open-ended questions to children to determine the kinds of computer games they engage in and then classified their responses. The predominant response (16.7% or 64 children) was "practical animal games." Additional comments included "educational games" and "intelligence games," in that order.

**Table 2:** Children's media device screen time by age

Age groups (months)	1-12 months (n=105)	13-24 months (n=141)	25-36 months (n=133)	37-48 months (n=178)	49-60 months (n=173)
Television					
None	39 (37.1%)	38 (27%)	8 (6%)	13 (7.3%)	3 (1.7%)
30 min-1 hour	43 (41%)	62 (44%)	53 (39.8%)	60 (33.7%)	66 (38.2%)
1-2 hours	20 (19%)	29 (20.6%)	52 (39.1%)	58 (32.6%)	57 (32.9%)
3-4 hours	1 (1%)	10 (7%)	11 (8.3%)	34 (19.1%)	34 (19.7%)
≥5 hours	2 (1.9%)	2 (1.4%)	9 (6.8%)	13 (7.3%)	13 (7.5%)
Computer					
None	96 (91.4%)	126 (89.4%)	117 (88%)	140 (78.7%)	133 (76.9%)
30 min-1 hour	4 (3.8%)	8 (5.7%)	7 (5.3%)	22 (12.4%)	14 (8.1%)
1-2 hours	4 (3.8%)	2 (1.4%)	8 (6%)	11 (6.2%)	17 (9.8%)
3-4 hours	1 (1%)	4 (2.8%)	1 (0.8%)	3 (1.7%)	8 (4.6%)
≥5 hours	0	1 (0.7%)	0	2 (1.1%)	1 (0.6%)
Tablet					
None	92 (87.6%)	122 (86.5%)	100 (75.2%)	104 (58.4%)	92 (53.2%)
30 min-1 hour	4 (3.8%)	11 (7.8%)	19 (14.3%)	33 (18.5%)	37 (21.4%)
1-2 hours	8 (7.6%)	5 (3.5%)	10 (7.5%)	32 (18%)	33 (19.1%)
3-4 hours	1 (1%)	2 (1.4%)	3 (2.3%)	4 (2.2%)	6 (3.5%)
≥5 hours	0	1 (0.7%)	1 (0.8%)	5 (2.8%)	5 (2.9%)

Upon evaluation, we observed that 69.8% of children's tablet usage (510 children) never engaged in play. One hundred and four children (14.2%) play with the tablets for 30 minutes to one hour, 88 children (12%) for one to two hours, 17 children (2.3%) for three to four hours, and 12 children (1%) for more than five hours (Table 2). The statistical analysis did not find a significant association (p>0.05) between having at least one sibling and the amount of time spent playing with a tablet. Children who received maternal care had significantly lower rates of tablet usage compared to children who received care from individuals other than their mothers (p<0.05).

There was no statistically significant disparity observed between genders in terms of time spent watching television, using computers, playing with tablets, or initiating television viewing (p>0.05). Although the proportion of children engaging in television viewing and computer and tablet usage is lower among those who do not attend kindergarten, it is noteworthy that nursery school attendees exhibit a higher prevalence of these activities (p<0.05).

The duration of nocturnal sleep in children is consistent across all age categories, with the majority sleeping for approximately nine to 10 hours. When asked about their daytime sleeping habits, 505 families (69.1%) reported sleeping during the day, while 226 families (30.9%) reported not sleeping during the day. The average daily sleep duration of 487 children who sleep during the day is 2.22±1.01 hours. Regarding the sleep patterns of children, 404 children (55.3%) sleep between 20:30 and 22:00, 271 children (37.1%) sleep between 22:30 and 24:00, 35 children (4.8%) sleep between 18:00 and 20:00, and 21 children (2.9%) sleep after 00:30. Comparing the bedtime and media usage periods of children revealed that children who went to bed before 22:00 had lower rates of television watching (p<0.05). There was no statistically significant correlation between the duration of nighttime hours and the amount of time spent playing on tablets or computers (p>0.05). The study found no statistically significant correlation between children's overnight electronic device usage and their sleep patterns in their own bedrooms (p>0.05). Youngsters who abstained from television viewing exhibited a greater tendency for daytime sleeping habits, whereas those who watched television for more than five hours did not display such habits (p<0.05).

When evaluating families' approach to media use and their level of control over their children's use, 433 individuals (59.2%) responded affirmatively, whereas 298 individuals (40.8%) responded negatively to the question "Do you control the tablet/computer your child is playing on?" A total of 451 individuals, accounting for 61.7% of the respondents, answered affirmatively to the query "Do you impose restrictions on your child's media consumption?" Conversely, 280 individuals, representing 38.3% of the respondents, responded negatively. A total of 28 individuals (3.8%) answered affirmatively, while 703 individuals (96.2%) responded negatively to the query "Has a social media account been created on behalf of your child?" Among the children with social media accounts, 10 have Instagram, five have Facebook, and five have both Facebook and Instagram profiles. Eight families affirmed their agreement with this question; however, they did not provide any further explanation. The rate of media restriction among graduate fathers, university graduate mothers, and their children is significantly greater compared to parents with lower education levels (p<0.05).

A comparison of children's computer and tablet usage time and their parents' behavior in monitoring the content watched by their children shows that children from families who closely monitor the content have more time to play games with these devices. On the other hand, children from non-controlling families play with computers and tablets less or not at all (p<0.05).

A total of 305 individuals, accounting for 41.7% of respondents, answered affirmatively to the query "Do you utilize a television or tablet while consuming food?" Conversely, 426 individuals, including 58.3% of respondents, responded negatively. A total of 315 individuals, accounting for 43.1% of the respondents, answered affirmatively to the query "Do you employ mobile phones or tablets for relaxation?" Conversely, 416 individuals, including 56.9% of the participants, responded negatively. Children beyond the age of two whose families used television or tablets while eating had a higher prevalence of this practice compared to children under the age of two, as observed. This difference was shown to be statistically significant (p<0.05). However, the practice of utilizing mobile phones to alleviate stress does not vary based on age (p>0.05). Furthermore, the practices of households utilizing mobile devices for television or tablet use and to pacify themselves during meals exhibit no significant variation between mothers and caregivers (p>0.05).

When asked about their understanding of pre-screen and front screen time, regardless of whether they had received guidance on screen front time or not, most individuals were questioned about their doctors' perspectives on screen front and back screen ideas and responded negatively (n=646; 88.4%). Regarding the question "Are you familiar with the term 'front of the screen'?", 631 (86.3%) answered "no". To the question "Is your child present in the room while you watch television?", 93.3% of respondents answered negatively.

The level of understanding of the notion of screen time is significantly greater among mothers with graduate degrees compared to those with primary, middle, and high school education (p<0.05). Families who demonstrated familiarity with the notion of front screen time were seen to impose greater restrictions on media usage, and this disparity was deemed statistically significant when compared to those who were unfamiliar with the concept (p<0.05). When analyzing the correlation between understanding the concept of screen time and the impact it has on children's behavior, it is evident that there is a statistically significant difference in the number of families who are unaware of this concept among children who watch television for more than four hours compared to those who are aware (p<0.05). No significant correlation was found between the duration of children's gameplay on a computer or tablet and the amount of time spent looking at the screen (p>0.05).

## Discussion

In this study, the number of digital screens is higher in homes and younger children's rooms. When examining the technical equipment present in the households of the children who took part in our study, we found that 98.6% had a television at home, 96.9% had a mobile phone at home, 54% had a computer at home, 49.5% had a tablet at home, and 34.1% had a gaming console at home. A study revealed that 13% of the children had a television in their rooms, while 11.9% owned a tablet, 7.4% had a computer, and 7% possessed a mobile phone. Around 65.8% of parents did not know of the concept of screen time, while 88.4% of parents did not know of the concept of back screen time. Screen exposure begins in the first months of life, and parents, especially those with children under the age of five, do not adhere to current screen time recommendations. Previous studies have consistently demonstrated that ease of access significantly contributes to the increased utilization of technological tools during the preschool period [33]. Kabali et al.'s study [20] found that 97% of children between the ages of six months and four years old had access to television at home, 83% had access to tablets at home, and 77% had access to smartphones at home. Additionally, 96.6% of children in the study had utilized these devices before reaching one year old [20]. Kılıç et al. discovered that 83.7% of children under the age of five owned their own tablets in their study on young children's exposure to portable displays. The utilization of tablets experiences a substantial surge following a period of 25 months [34].

In the United Kingdom, 75% of these infants aged 6-36 months begin watching television within the first 12 months of their lives, and this viewing time progressively increases as they grow older. Specifically, at 14 months, children watch less than one hour per day, but by 30 months, their daily television consumption exceeds two hours [35]. An Italian study revealed that 20% of children initiated smartphone usage before their first birthday, while 80% began using their parents' phones between the ages of three and four [36]. Chang et al. [37] found that among children aged 2-5, 65% started watching television before the age of two, 12.2% used a smartphone before the age of one, and 31% used a smartphone before the age of two [37]. Our study found that 75.9% of children initiated television viewing before the age of two. These findings emphasize the importance of educating parents about evidence-based guidelines for reasonable media consumption.

In parallel with the increase in the number of televisions in the home and in children's rooms, children's television viewing time has also increased over the years. Carson and Janssen [38] conducted a study focusing on children under the age of five, and it showed that 13.6% of the children spend over two hours daily whereas 43.5% spend over one hour daily watching television. In our study, 86.2% of children younger than five years watched television for at least 30 minutes a day, and 37% of these children were younger than two years. Our study of daily television viewing habits revealed that a significant proportion of parents (43%) spent one to two hours watching television. Most children under two years of age who watched television watched more than one hour. In 2013, the Bernard van Leer Foundation conducted a detailed survey among 4101 children aged 0-8 in Türkiye, and it showed that a significant number of children (23.6% in the age group of 0-2 and 70.6% in the age group of 2-5) spend more than two hours daily watching television [39]. There is a correlation between the amount of time families spend watching television and the amount of time children spend watching television [33,40]. A study involving 2965 Portuguese children aged 3-10 years revealed a robust correlation between the television viewing habits of families and children across all age categories, irrespective of gender. Mothers' television viewing time has a greater impact on children's television viewing time compared to fathers [33]. In our study, family member's longer watching time was associated with longer children's watching time. Therefore, in determining watching television time in children, it would be useful to regulate the television viewing habits of family members at home.

Research suggests that children whose mothers have a lower level of education tend to experience delays in their cognitive development [21]. Some previous studies showed that there is an inverse relationship between the duration and level of education of the mother and the duration of television viewing of the child [41,42]. Furthermore, some studies, like our study, fail to show a substantial correlation between the educational status of the family and the amount of time children spend using screens [6]. The presence of an older child in the household has an impact on the amount of time the younger sibling spends using the internet and screens [1,43]. Older siblings are also inclined to promote the use of digital media among younger siblings [44]. We showed that there has been a substantial increase in the amount of time they spend watching television and playing computer games among children who have at least one sibling. The correlation between having siblings and increased screen exposure time suggests the necessity for parents to establish and enforce a unified and logical media usage policy for all children residing in the household.

Families and children commonly use digital screens for many purposes: communication, viewing photographs, sharing photos, homework, and researching information [45]. According to the study conducted by Wu et al., children use digital technology for various purposes, including 75% for literacy, 72% for sound and color recognition, 58% for completing school assignments, and 58% for enhancing memory [5]. Research has demonstrated that children exhibit a greater preference for educational games that stimulate cognitive abilities, virtual reality games, and racing games compared to other types of games [46]. In our study, 38.8% of parents reported that their children predominantly watched cartoons on television. The study found that news programs and television series were the most watched, followed by music channels at home.

Television viewing can contribute to obesity through many mechanisms [47]. These factors are a sedentary lifestyle and decreased physical exercise, the influence of unhealthy food choices promoted through commercials, increased snacking habits while watching television, and disrupted sleep patterns [48]. The majority of the advertisements are food-related and specifically promoting sugar and sugary foods. Individuals who spent more time watching television on weekends had an increase in BMI at the age of 30. Each additional hour of television viewing over weekends at the age of five is associated with a 7% higher chance of developing obesity in adulthood [49]. Wen et al. [50] showed a direct correlation between the amount of time spent consuming media and a rise in BMI. In our study, out of the entire sample, 24.9% had protein-energy malnutrition, and 5.3% were obese. We found a correlation between the duration of screen exposure starting at the age of two and the BMI which suggests the need to educate families about the potential long-term effects of screen usage.

There is a strong link between media use and sleep in school-age children and adolescents, especially with delayed bedtime and/or lower total sleep time [51]. Technological devices are significant contributors to the emergence of sleep issues in children [52-54]. Engaging in both active (playing games) and passive (sitting in the room with the television on) television viewing between the ages of five and six can lead to sleep-wake transition difficulties, difficulty sleeping, and overall sleep disorders [54]. A study revealed that each additional hour of daily television viewing resulted in a drop of seven minutes in sleep duration. Furthermore, having a television in the bedroom led to a reduction in sleep time ranging from eight to 31 minutes [27]. We showed a correlation between screen exposure and a reduced duration of daytime sleep, and an inverse relationship was detected between screen exposure and the duration of sleep during the night. We hypothesized that there was a notable disparity in the occurrence of televisions, computers, and mobile phones in children's bedrooms and their impact on their sleep duration at night and daytime sleep patterns. A study conducted in Korea examined the 7-9 and 10-12 age groups and discovered that prolonged smartphone use led to a decrease in blinking movement, resulting in dryness of the eyes. Furthermore, the proximity of smartphone usage leads to ocular fatigue, glare, and discomfort [55]. In our study, we identified 44 individuals (6%) with visual defects. However, our analysis did not reveal any statistically significant association between these visual impairments and the amount of time spent watching television.

Our study has demonstrated a positive correlation between the age of children and their utilization of media tools for eating and calming purposes. In Australia, families reported that they allocate 44% of their resources towards promoting tranquility and happiness in their children, 37% towards education, and 20% towards communication. Sixty percent of them utilize these devices at home, while 22% use them while traveling, and 17% use them outdoors [56]. Kılıç et al. [34] showed that 59.6% of families permit their children to use mobile gadgets when performing their daily tasks, whereas 28.8% of families grant permission during shopping [34]. In a study conducted by Jago et al. [33], including children aged three to six years attending kindergarten, it was found that 48.7% of the children reported watching television while eating. Our study revealed that the prevalence of families utilizing television or tablets during meals was 47% in children aged two years and older and 31.3% in children under the age of two.

The duration spent engaging with various digital media platforms, including television, computers, mobile phones, and tablets, encompasses screen front time [35]. Our study revealed that the majority of parents (65.8%) are unfamiliar with the notion of screen time. Around 88.4% of families are unfamiliar with the idea of back screen time, which refers to the duration spent in a room while any screen is active. The percentage of children present in the room during family television viewing is 86.3%. Exposure to background television has adverse effects on children's playtime, parent-child connection, and cognitive task performance [6]. A study from the United States showed that, on average, each child spent 232.2 minutes per day with television in the background. The prolonged duration of the back screen front time resulted from leaving the television on while unattended and the children entering the bedrooms [57]. Our study revealed that families who were familiar with the notion of screen time implemented stricter limitations on media usage. Conversely, families with children who watched television for more than four hours showed a lower level of awareness regarding this concept. It demonstrates the importance of families possessing information about these two topics. There is currently no correlation between the duration of children's computer and tablet usage and the concept of front screen time.

The AAP advises against exposing children to digital media until the age of 18-24 months. Families who wish to introduce television to children aged 18-24 should opt for high-quality shows and watch them together while selecting age-appropriate programming for children aged two to five and limiting viewing time to one hour. While observing, they propose that they comprehend what they perceive and assist them in recognizing their comprehension. Simultaneously, they suggest removing screens from the children's bedrooms and deactivating all devices one hour before bedtime [15]. Asplund et al. [25] revealed that 53% of the children adhered to the AAP guidelines on screen time, 56% followed the AAP's advice on keeping bedrooms free of televisions, and 29% of the children were not in their bedrooms but still complied with the screen time recommendations. Adhering to the recommendations becomes more manageable when families restrict television viewing at home to fewer than two hours, switch off the television during meal times, and allocate less time to using media devices [25]. AAP advises clinicians to inquire about the amount of time families and their children spend on screens during their initial interaction. Parents should prioritize the significance of engaging their children in social games in order to foster early brain development and enhance language, cognitive, and socioemotional skills. Consequently, they should regulate their children's screen time based on their age, opting for high-quality programs that are age-appropriate. It is particularly beneficial to engage in educational activities together, especially before the age of five [15]. Dinleyici et al.'s [32] previous study revealed that families possess insufficient understanding regarding screen time and sensible media usage. Consequently, we recommend providing evidence-based recommendations on this matter. According to our research, 93.3% of the families reported that their doctors had not previously provided them with information regarding their screen usage. In family medicine and child health outpatient clinics, where the monitoring of healthy children takes place, it is important to incorporate the appropriate use of media into the recommendations given to parents. This information should be considered as part of preventive health services for the new generation growing up in the digital age. It is crucial to allocate sufficient time during the visit to discuss these recommendations.

Limitations

Our study had some limitations. It was conducted in a single center, and the results were interpreted according to the parent's own statements. The health status (mainly physical) of the parents was noted according to their own statements and physical health characteristics. Our study belonged to the pre-pandemic period. In order to mitigate the transmission of coronavirus disease 2019 (COVID-19), several measures were implemented, including the closure of schools, mandatory isolation, adherence to social distancing guidelines, and the cancellation of social activities [58]. The COVID-19 pandemic has caused significant disturbances in the lives and daily schedules of children, adolescents, and families, resulting in a probable rise in the amount of time spent using electronic screens. The latest meta-analysis conducted by Madigan et al. examined 46 research and found a significant rise in screen time, namely, by 52%, primarily among teens [58].

## Conclusions

The number of digital screens is increasing in homes and children's rooms. Screen exposure starts in the first months of life, and current screen time recommendations are not followed, especially in children under the age of five. The fact that children with siblings have more screen exposure time indicates that parents need to develop and implement a common rational media usage rule for all children in the home. Families should be informed about the long-term consequences of screen exposure, as the relationship between screen time from the age of two and BMI suggests. Families should receive information about evidence-based advice on rational media use. Rational use of media and information to inform parents about treatment and care in parents and child health and disease outpatient clinics where healthy child surveillance is performed should be accepted as a preventive healthcare service for the new generation growing in the digital age.
